# New purple sulfur bacteria genomes from Nebraska salt marsh enrichments

**DOI:** 10.1128/mra.00927-26

**Published:** 2026-09-10

**Authors:** Maria F. Orta Medellin, Evelyne Y. Aguirre Zepeda, Genesis Gallegos Martinez, John A. Kyndt

**Affiliations:** 1 Instituto de Ciencias y Estudios Superiores de Tamaulipas A.C. Campus Tampico 2000 (ICEST), Tampico, Tamaulipas, Mexico; 2 College of Science and Technology, Bellevue University 52617, Bellevue, Nebraska, USA; Montana State University, Bozeman, Montana, USA

**Keywords:** purple bacteria, Nebraska salt marshes, hydrogenase-4 operon, *Halochromatium*, *Ectothiorhodospira*

## Abstract

Two genomes from a Winogradsky column enrichment from the Nebraska salt marshes were sequenced. The analysis of whole-genome phylogeny and average nucleotide identity comparisons indicated that these belong to species of purple sulfur bacteria, *Halochromatium* and *Ectothiorhodospira marina*, that have not been described before.

## ANNOUNCEMENT

The Nebraska salt marshes are a rare inland ecosystem that is fed by salty groundwater, which creates a unique plant, insect, and microbial biodiversity (1–4). Several unique bacterial species have been identified through seasonal metagenomic analysis in the past few years (3, 4). However, most of these have not been able to be cultivated in the lab. We now performed an enrichment of the salt marsh microbes in a Winogradsky column to identify and isolate more unique photosynthetic species. The column (10 cm × 4 cm) was placed under daylight illumination in front of a window for 4 weeks. The sample material for the Winogradsky column was obtained from the following location: latitude, 40 52′54.42″ N; longitude, 96 39′35.82″ W. Samples were taken using 100 mL sterile bottles and stored overnight at 4°C until the Winogradsky column was assembled the following day and placed in the light at 25°C.

A dark red band appeared on the Winogradsky column after 4 weeks of incubation at about 2 cm depth, and 1 mL of sludge was taken by slow pipetting near the glass edge of the column. This was used for genomic DNA extraction using the GeneJET DNA purification kit (Thermo Scientific). This resulted in DNA with a concentration of 68.3 ng/μL and an A260/280 of 1.85. The sequencing library was prepared using the Illumina DNA Library Prep kit and sequenced by an Illumina MiniSeq using 500 μL of a 1.8 pM library. Paired-end (2 × 150 bp) sequencing generated 2,679,692 reads and 399.3 Mbps. Quality control of the reads was performed using FASTQC (v1.0.0), using a k-mer size of 5 and contamination filtering for overrepresented sequences against the default contamination list. Illumina adapter sequences were removed using the FASTQ Toolkit (v2.2.6) within BaseSpace (Illumina). The genome was assembled using the Metagenomic binning function in BV-BRC (5, 6), which uses MetaSPAdes (v3.12.0) (7). This provided two good quality bacterial genomes of 3,584,162 bp (63.7% GC) (NSM3_A) and 3,548,302 bp (62.1% GC) (NSM3_B). The genome characteristics are summarized in Table 1. The final genomes were >99% complete according to CheckM (v1.1.6) (8). An EvalG genome quality analysis as part of CheckM showed <2% (NSM3_A) and <4% (NSM3_B) potential contamination. Genomes were annotated by NCBI PGAP (v6.6) (9). Default parameters were used for all software applications unless otherwise noted.

**TABLE 1:** Overview of genome features of the related sequenced *Halochromatium* species and *Ectothiorhodospira* species compared to the two newly sequenced strains^*a*^

Species	Size (Mb)	% GC	Contigs	Coverage (×)	N50	CDS	tRNAs	ANI	Reference	GenBank accession #
*Ectothiorhodospira* sp. NSM3	3.6	62.1	162	111	62,990	3,419	43	–^*b*^	This paper	JCAQHZ000000000
*Ectothiorhodospira marina* DSM 241 [T]	3.2	62.3	41	378	143,237	3,059	44	97	(10)	FOAA00000000
*Ectothiorhodospira haloalkaliphila* 9902	3.7	62.7	243	106	157,842	3,602	44	86.6	(11)	JAJNQR000000000
*Ectothiorhodospira mobilis* DSM 237 [T]	2.8	68.3	119	539	77,281	2,714	44	75.7	(10)	NRSK01000000
*Halochromatium* sp. NSM3	3.6	63.7	103	112	78,675	3,540	44	–	This paper	JCAQIA000000000
*Halochromatium salexigens* DSM 4395 [T]	3.8	63.7	100	61	66,939	3,831	46	89.2	(10)	NHSF00000000
*Halochromatium roseum* DSM 18859 [T]	5.2	62	88	50	143,744	5,208	44	80.7	(10)	NHSH01000000
*Halochromatium glycolicum* DSM 11080 [T]	5.3	64.6	147	75	84,551	5,291	45	78.6	(10)	NRSJ00000000

^*a*^ The new genomes are indicated by gray shading. Average nucleotide identity (ANI) percentage is based on bidirectional ANIb values to strains NSM3-A and NSM3-B, calculated using JSpecies.

^*b*^ –, reference genome for ANI calculations.

**Fig 1:**
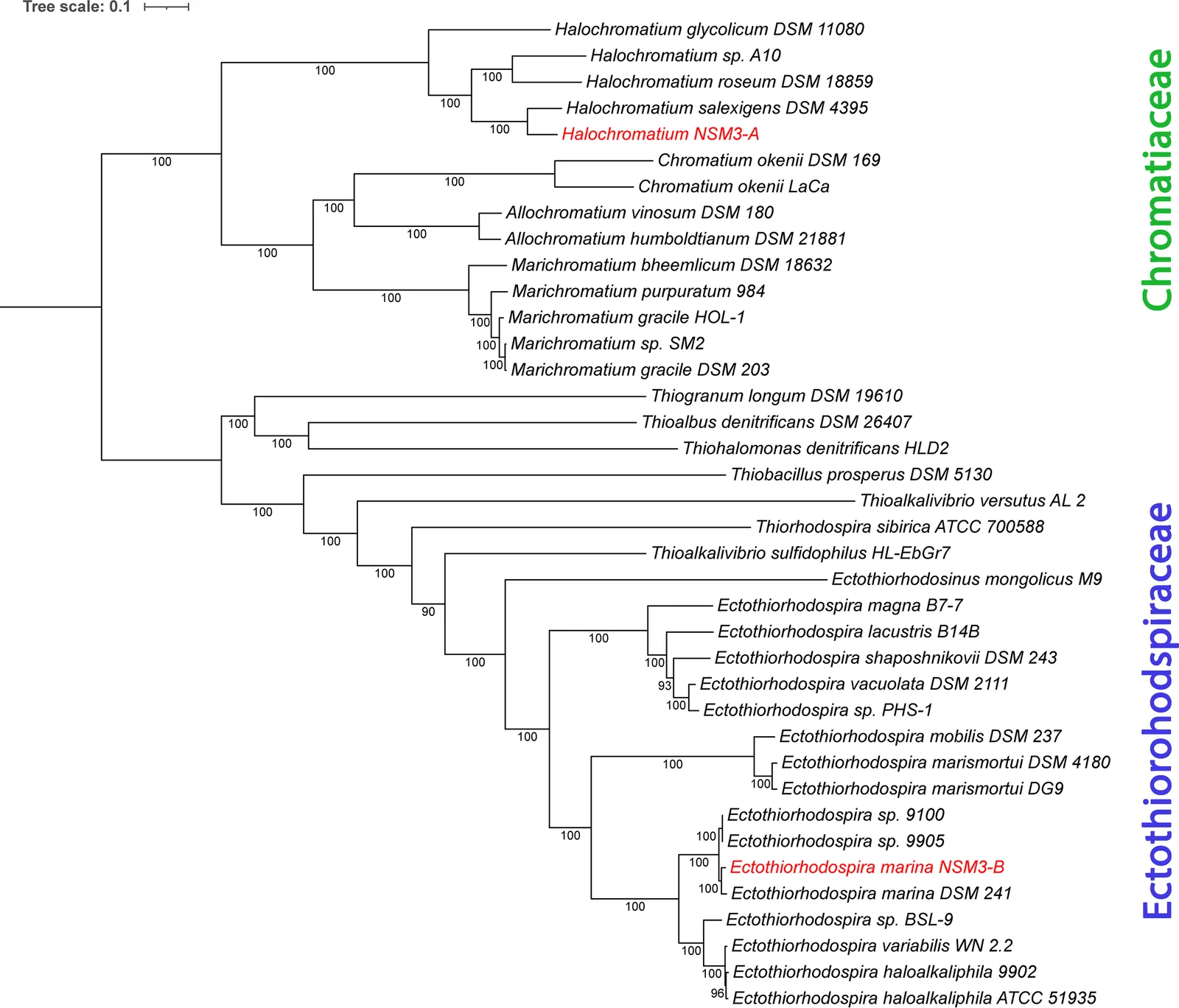
Phylogenetic tree of *Halochromatium* NSM3-A and *Ectothiorhodospira* NSM3-B whole-genome comparison to their closest relatives. The phylogenetic tree was generated using the Codon tree method within BV-BRC (7), which used PGFams as homology groups and analyzed 141 aligned proteins and coding DNA from single-copy genes using RAxML (v8) (13, 14). The support values for the phylogenetic tree are generated using 100 rounds of the “Rapid bootstrapping” option of RAxML. The tree was midpoint rooted, and iTOL was used for the tree visualization (17). Species with new genomes are indicated in red, and the taxonomic family name is indicated in blue and green.

A JSpecies comparison (v4.1.1) (12) of ANI percentage ranged from 89.2 to 78.6 for strain NMS3_A with *Halochromatium* species (Table 1) and showed 97.0% identity of strain NSM3-B with *Ectothiorhodospira marina*. Phylogenetic comparison of the two new genomes to the other known *Ectothiorhodospira* and *Halochromatium* relatives in NCBI GenBank using RAxML (13, 14) showed *Halochromatium salexigens* DSM 4395 as the closest relative to genome NSM3-A, while *Ectothiorhodospira marina* DSM 241 as the closest relative for NSM3-B (Fig. 1). Strain NSM3-B may be a new strain of *Ectothiorhodospira marina*; however, the ANI of all the *Halochromatium* species is far below the arbitrary species cutoff (of 95%), indicating that strain NSM3-A might belong to a novel species. Further isolation and physiological characterization experiments are underway to formally propose a new species. Interestingly, a proteome comparison analysis in BV-BRC showed that *Halochromatium* NSM3-A contains 116 unique PGFams compared to the other *Halochromatium* species and is the only one that contains a hydrogenase-4 operon, which is thought to play a critical role in anaerobic energy metabolism by catalyzing the breakdown of formate into carbon dioxide and hydrogen gas, particularly tailored for survival in harsh conditions such as energy stress and extreme pH (15, 16).

## Data Availability

This whole-genome shotgun project has been deposited at DDBJ/ENA/GenBank under Bioproject PRJNA1489541 with the accession numbers JCAQHZ000000000 (*Ectothiorhodospira marina* NSM3) and JCAQIA000000000 (*Halochromatium* sp. NSM3). The raw sequencing reads have been submitted to SRA, and the corresponding accession number for the Illumina data is SRR39850531.
